# Artificial intelligence in medical imaging practice in Africa: a qualitative content analysis study of radiographers’ perspectives

**DOI:** 10.1186/s13244-021-01028-z

**Published:** 2021-06-16

**Authors:** William Kwadwo Antwi, Theophilus N. Akudjedu, Benard Ohene Botwe

**Affiliations:** 1 Department of Radiography, School of Biomedical and Allied Health Sciences, College of Health Sciences, University of Ghana, Korle Bu, Box KB143, Accra, Ghana; 2 Institute of Medical Imaging and Visualisation, Department of Medical Science and Public Health, Faculty of Health and Social Sciences, Bournemouth University, Bournemouth, UK

**Keywords:** Artificial intelligence, Medical imaging, Radiography, Africa, Online surveys, Qualitative study

## Abstract

**Purpose:**

Studies have documented the clinical potentials of artificial intelligence (AI) in medical imaging practice to improving patient care. This study aimed to qualitatively explore the perception of radiographers relating to the integration of AI in medical imaging practice in Africa.

**Methods:**

The study employed a qualitative design using an open-ended online instrument administered between March and August 2020. Participants consisted of radiographers working within Africa during the time of the study. Data obtained were analysed using qualitative content analysis. Six themes of concerns were generated: expectant tool; career insecurity; cost of new technology, equipment preservation and data insecurity; service delivery quality; need for expanding AI awareness.

**Results:**

A total of 475 valid responses were obtained. Participants demonstrated a positive outlook about AI in relation to clinical quality improvement, competent diagnosis, radiation dose reduction and improvement in research. They however expressed concerns relating to the implementation of this technology, including job security and loss of core professional radiographer skills and roles. In addition, concerns regarding AI equipment maintenance, lack of awareness about AI and education and training opportunities were evident.

**Conclusion:**

Awareness of the importance of AI in medical imaging practice was acknowledged; however, concerns relating to job security, data protection must be given critical attention for successful implementation of these advanced technologies in medical imaging in Africa. Inclusion of AI modules in the training of future radiographers is highly recommended.

**Supplementary Information:**

The online version contains supplementary material available at 10.1186/s13244-021-01028-z.

## Key points

- Radiographers working in Africa reported their willingness to accept artificial intelligence (AI) in medical imaging practice.
- The African radiography workforce is concerned about job security and lack of knowledge regarding AI.
- AI-related training programmes and roadmaps for its implementation in Africa are urgently needed.

## Background

Healthcare is quickly evolving, and current technological developments are centred largely on the increasing integration of complex computerised algorithms into equipment modalities [1–3]. Artificial intelligence (AI) is a key component of these complex algorithms and is currently applied innovatively in healthcare because of its reported advantages and the potential to improve patient care [2, 4]. AI has been widely used in many clinical circumstances to diagnose, treat and predict the outcomes [5]. Specifically, healthcare and research applications include prediction of disease prognosis and response to treatment, drug development, remote patient observations, medical data management, digital patient consultation and in some cases administrative hospital management [6–8].

In medical imaging practice, AI has shown impressive accuracy and sensitivity in the identification and characterisation of abnormalities leading to enhanced service delivery and quality of patient care [9]. Increasing developments of the technology has propelled its application beyond image interpretation in medical imaging practice. Currently, AI systems are employed across equipment modalities to improve image acquisition and quality of care [1, 10, 11]. The impact of AI in medical imaging has been summarised in a publication by Lewis and colleagues [4]. They indicated that AI would lead to workflow improvement, production of high-quality images, improvement in image interpretation, image segmentation, automated image registration and radiomics analysis and dose optimisation, automate scheduling and protocolling and reduce scanning times. These would improve the work of radiographers, radiologists and other medical imaging staff and help deal with workloads which have led to staff shortage. It is indeed thought to revolutionise the entire imaging and healthcare industry in the near future. Studies are exploring the use of AI in the provision of human trait-related solutions like empathy and companionship to enhance quality of life through systems *like “Vits”* [12]. This has instigated arguments about the roles and responsibilities of healthcare professionals including radiographers.

Of note, referring physicians will have responsibilities as well, since they decide on therapeutic management based on the imaging report (and often multidisciplinary team discussions), including evaluation on early response on therapy. In addition, many (minimally invasive) current interventional procedures are image-guided, based on AI-processed images and fusion of different modalities. As a result of the broad areas that AI could be useful, it is anticipated AI would greatly bear on the radiologist’s regular routine and improve practice, but it would not entirely change their core practice [9].

Notwithstanding, the medical community must anticipate the potential unknowns and professional requirements of this technology to ensure effective, continuous and safe incorporation into diagnostic imaging practice [1, 10, 13]. This has initiated arguments about the roles and responsibilities of imaging professionals who will use this technology, particularly, radiographers and radiologists [13, 14]. Lewis and colleagues [4] reported that some radiographers may have a scary or exciting perception about AI, and these could be heightened by the thought of having an ‘AI work colleague’ in the radiology department. This clearly suggests that AI systems may need to fulfil certain conditions to be fully embraced by health professionals and the society [15].

To understand the views and readiness of radiographers on the use of AI in medical imaging practice, some studies have been conducted [16–18]. These studies explored the perspective of radiographers on the integration of AI into medical imaging practice to identify factors that could help improve the implementation process. However, these studies mostly employed quantitative approaches, with associated methodological limitations relating to the depth of perspectives provided. To the best of our knowledge, there is rarity of studies employing qualitative designs to explore perspectives on AI in medical imaging practice. Radiographers serve as the interface between technologies and their patients [14]; thus, this study qualitatively explored the perceptions of this workforce on the use of AI in practice in Africa—a low-resource setting. Qualitative opinions about new developments could be predictable; thus, it is expected that radiographers as end users would have some concerns about AI. However, radiographers in Africa would have unique concerns due to the resource challenges they deal with at work.

## Methods

The study is a sequel to a quantitative study [18] which concluded with an open-ended question for participants to share their views about AI. Purposive sampling was used as it is the most appropriate approach for content analysis inquiry [19]. The sample used consisted of radiographers across the five geographical regions of Africa with different cultural and linguistic backgrounds. To ensure anonymity of the participants, the radiographers were assigned pseudonames such as “Rad 01”. In the COVID-19 pandemic, semi-structured face-to-face interview was not possible and to maximise response across multiple countries in Africa, open-ended online surveys were employed for this study.

This exploratory cross-sectional online survey was hosted on Google Forms (Google, Mountain View, CA). The instrument broadly included questions relating to demographics, general attitudes, and perspectives on AI and how it should be implemented in Africa, job security, the future of medical imaging including workforce development and ethics in relation to the integration of AI (Additional file 1: Appendix 1- Questionnaire). The free text comments provided in response to the open-ended question “any other comments” are of interest because information provided by such open-ended questions is used to substantiate answers to a structured questionnaire [20]. The use of open-ended questions provided study participants, the chance to express their judgements about an issue [20]. Qualitative survey studies are essential for determining the diversity of opinions, perceptions and experiences on a topic of interest within a given population [21]. The non-anglophone responses (Arabic and French) were translated into English by bilingual academics who are radiographers and members of the research team. No transcription was done because participants provided textual response. To ensure a robust analysis and interpretation, the obtained data were collaboratively analysed by three researchers (W.K.A., B.O.B., T.N.A.) using qualitative content analysis—a recommended approach for the analyses of textual data [22]. Trustworthiness for the content analysis was ensured using established criteria [23]. They argue that the purpose of using trustworthiness in a qualitative study is to back the argument that the research outcome is “worth paying attention to”.

### Ethical consideration

The Ethics and Protocols Review Committee of the School of Biomedical and Allied Health Sciences of the University of Ghana granted approval for the study (SBAHS/AA/RAD/29245/2019–2020).

## Results

**Fig. 1:**
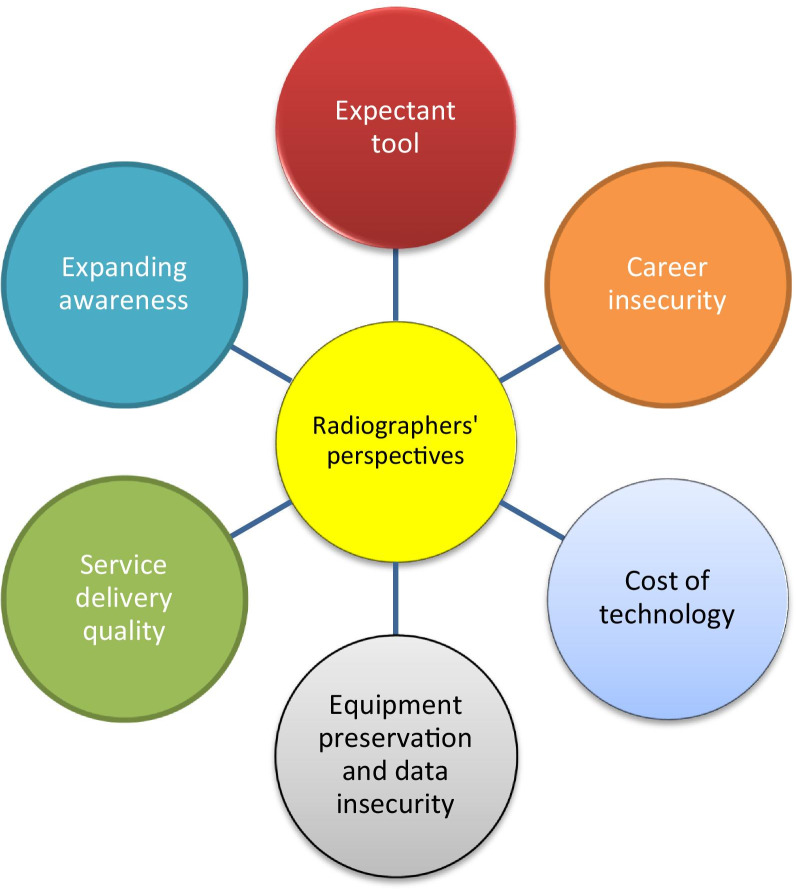
Summary of radiographers’ perspectives on AI technology implementation and usage

A total of 475 respondents provided qualitative responses out of the 1020 who took part in the original quantitative study [18]. Of the 475 comments which have been analysed for the current study, the majority (360) were males and 115 females. Although respondents held a broad range of educational qualifications (from certificate to Ph.D.), the majority held Bachelor of Science or Technology degrees. The most common age group of the respondents was 30–39 years old. The combined analysis of information derived led to the generation of six themes about AI integration in Africa including expectant tool, career insecurity, equipment preservation and data insecurity, service delivery quality, cost implications of AI technology and the need to expanding AI awareness. Figure 1 shows a summary of radiographers’ general perspectives on AI implementation and usage.

### Expectant tool

Most of the respondents accepted AI as a promising and inevitable tool for Africa. Although a new technology is yet to take root in Africa, many of the participants were optimistic about AI.

AI is here to stay. We need to embrace it. (Rad 2)

This is new concept in Africa. With more evidence on application of the tool, more departments are likely to embrace the technology. A perceived likely challenge is the reduced human interaction with the patient hence reduced psychological aspects of care. (Rad 8).

AI technology is very important tool in Africa (Rad25).

I think AI will nourish imaging in Africa (Rad 26).

The intervention of AI in Africa will be an excellent innovation because of the enormous advantages in AI (Rad 32).

I think this consideration of AI in medical Imaging opens up great possibilities for growth. (Rad 15)

I think AI will be a revolutionise Radiology (Rad 29)

While several of the participants are of the view that AI will lead to progress and improvement of medical imaging practice, as evidenced above, others felt otherwise.

I don't think it’s not ok for AI to come to Africa, we are going to face a lot of mistakes in health facility when using those tools. (Rad 53).

AI not safe in radiology department due to lack nuclear security. (Rad 54).

One participant (Rad 290) was comfortable with the old school (the usual conventional radiographs). While some expressed knowledge gap about the technology.

I love radiograph (Rad 29)

We need more knowledge about AI, as for me personally I don't know anything about this. Thank you. (Rad 419)

Critical analysis should be done before implementation. (Rad 442)

### Equipment preservation and data insecurity

A few of the participants were particularly disturbed about the substandard safeguarding of equipment in Africa, data security and software exploitation which to them should be given critical attention before AI is implemented.

For Africa maintenance culture is poor so this should be amongst top priority to be put into consideration to help ensure accuracy of results and promote longevity and management of these. Engineers and servicing staff should also be on hand to ensure smooth and accurate functioning of the equipment. Adequate training and resources required too before embarking on this. Should not replace the staff but rather enhance their job and promote efficiency of their results. (Rad 566)

AI is an unavoidable technology. However, the developers of the tools should emphasize more on management of the data it'll generate. I actually feel more concern about the big data and its security. (Rad 908).

AI would improve efficiency by eliminating human errors but my fear is software corruption which may be fatal to patient care. (Rad 96)

The maintaining of equipment is the only problem. (Rad 85).

### Service delivery quality

Participants were very hopeful that AI will improve practice quality, delivery and patient safety. Participants wrote about delivery of general efficiency of practice, competent diagnosis, and particularly AI could help physicians in selecting patients for the most appropriate imaging modality ('clinical decision support'), thus contributing to reduction of waiting lists, anxiety, dose and cost.

Al would be a welcome development in the field of medical imaging for more precise imaging techniques and diagnostic accuracy for better patient's management. (Rad 19).

AI in Africa will improve efficiency and accuracy in providing diagnostic medical services and enhance safety of patients! (Rad 28)

…radiology would no longer miss any pathology hence quality is improved and patients’ life would be saved. (Rad 34).

AI will greatly improve the quality outcomes and services of Radiographers in Africa; I truly believe that it will make the work much easier and interesting. (Rad 35)

AI in radiography could play a great role in our daily practices and help shorten waiting times for patients… (Rad 30).

…it will surely help in optimizing radiation dose to patient and also reduce radiologist reporting time. (Rad 33).

### Cost of new technology

A few of the participants were more concerned about the cost of the AI technology looking at the very challenging economic situation in most of Africa and whether the use of AI would lead to 'less studies' in 'less patients' as shared by some participants:

One area not covered is the initial costs involved in implementation and roll-out. (Rad 81)

The African continent is a mix of first world and third world conditions and, financially, may not be able to embrace the latest and greatest medical advances wholesale (Rad 882)

However, one participant had a different view on cost of the technology.

AI is a very important tool in medical imaging, as it is in other fields, it's the way forward to aid accurate diagnosis as well as reducing cost of expensive manpower. (Rad 82)

### Career insecurity

In the past, the radiology profession survived other major developments; one of the most striking was the development and implementation of the PACS environment, which in its early days was considered as the end of profession. This did not affect the job security but rather aided growth and service delivery and subsequently quality patient care. However, the majority of participants felt that AI will bring about job losses.

AI is great but a lot of people will be out of jobs. (Rad 5)

The integration of AI in Africa is good, but I think that it will affect Job security of the Radiographers and Radiologist… (Rad 19)

Integration of artificial intelligence in medical imaging is welcome development but may likely lead to unemployment for Radiographers and Radiologist. (Rad 22)

AI should undergo thorough investigations to avoid job loosing for many Radiographers. (Rad 103).

Further misunderstanding about AI and job insecurity was extended to the likelihood of the collapse of radiography as a profession.

For me I don't support this AI thing because it will leave most of us jobless and above all, radiography will be a useless course. (Rad 108)

Should not replace the staff but rather enhance their job and promote efficiency of their results. (Rad 75)

One participant was of the view that AI would revise the roles and the self-directed tasks of the radiographer and radiologists alike and as such more research into AI technology should be of priority before accepting the technology.

Not only will AI drastically reduce the autonomous tasks of both radiographers and radiologists, it will advantageously redefine their roles in future. (Rad 31).

Critical analysis should be done before implementations. (Rad 59)

AI is relatively a new concept in Africa. Needs more research and proper implementation. (Rad 72)

While some expressed concerns about the possibility of job lose others felt otherwise about their career and medical practice generally.

AI is coming and we must prepare to embrace it than to focus on the negative aspects such as - it may take away our jobs. Noooo! We choose how AI should operate. When we program it for good, excellent? However, when we program AI for bad then we shall surely face the consequences. (Rad 16)

AI in medical imaging will turn medical practice around for good. (Rad 18)

### Expanding AI awareness

AI was seen as a new technology that will require special training and/or education in addition to the radiography education received previously for conventional medical imaging. As a result, some felt the urgent need to improve users’ responsiveness to the new technology through education, training and more research before its implementation.

We need to get educated and trained. (Rad 110)

Before integrating AI, system dealing with lack of knowledge is more important (Rad 116)

AI is relatively a new concept in Africa. Needs more research and proper implementation. (Rad 73).

## Discussion

The current study is a follow-up to a previous quantitative inquiry [18], and qualitatively explored in-depth, the perception of radiographers in Africa about AI implementation in practice. Although the quantitative aspect [18] had 1020 respondents, only approximately half (*n* = 475) provided valid responses to the open-ended survey questionnaire. This was an opportunity for participants to provide unrestrictive views about what they perceived about AI implementation and usage in Africa.

Participants claimed that they were aware about AI (although their actual levels of awareness could not be ascertained) which is yet to gain grounds in Africa and had very positive expectation for the technology to change the traditional mode of medical imaging practice. This is consistent to the views reported in previous study from Ghana where 86.1% of the study participants expressed an awareness of AI in medical imaging practice [17]. Participants in the current study also shared that considering the numerous advantages, AI was certain to be the right technology for use in Africa. Besides they were confident that AI would bring about several innovations and growth in medical imaging practice and beyond (as it is already doing in many disciplines in medicine such as nuclear medicine, pathology, laboratory, genetics). Despite this positive stance, some participants accepted AI with mixed feelings, with concerns that the workforce may be affected negatively. Interrogations about AI application seem to cloud the minds of various healthcare institutions [24]. Many African countries are still using conventional radiographs with wet-image processing techniques and no wonder one participant expressed an attachment to the usage of conventional radiographs and would not probably like to see any change. A seeming misunderstanding centred on the possibility of reduced professional–patient interaction which to some might affect the psychological aspects of care. This claim is somehow doubtful as AI will not take away the radiographer–patient interaction opportunities that aid in understanding a patient’s psychological state (e.g. level of pain) and tailor care to offset any unusual emotions. On the contrary, the radiographer’s work involves direct patient interaction [25]. Patients would still need to be positioned by the radiographer for a procedure which provides the opportunity for the radiographer to communicate with the patient before the examination. The radiographer is obliged to pay attention to the psychological state of the patient to understand the diverse emotional states to inform patient-centred care for better outcomes [26].

As previously reported [17], participants in the current study also indicated that AI was not appropriate for Africa because these tools will lead to diagnostic medical errors, considering the associated margins of error with all mechanical systems. This appears to be an erroneous perception. Evidence suggests that the AI tools would rather improve the practice of the profession including high-quality diagnosis and minimal errors [2, 4, 5, 27]. A study [27] also indicated that AI tools provide better diagnostic decisions (thus, it takes into consideration the results from every medical technical result, e.g. radiology, nuclear medicine, pathology and others to produce a single and personalised diagnostic report). They also improve treatment outcomes and reduce medical errors, thus making it easier for medical professionals to care for a larger number of patients. For example, several AI-assisted diagnostic equipment currently has advanced cancer identification and detection functionalities, particularly in areas such as screening mammography, lung cancer screening and histopathological assessment of breast images [4]. This example leads to the argument that AI has certainly improved medical imaging, by demonstrating remarkable precision and sensitivity in the identification and classification of anomalies leading to improved care [9]*.* In medical imaging practice, AI could immensely improve the contribution of radiographers to processing bulky images, as for instance, chest radiographs. Even a simple AI-based tool to differentiate 'normal' from 'abnormal' could be helpful to reduce the radiologist's reporting workload and improve significantly radiological reporting (e.g. in depth and broadening even beyond the clinical context or radiological question) or particularly assist radiographers in rural and other low-resource settings where radiologists are not available. It must be emphasised also that the role of the radiologist will not change totally in the era of AI. The radiologist will be the responsible 'validator' of the radiological report, whatever additional AI-based tools are used.

Participants raised concerns about poor equipment maintenance culture in Africa which to some might not make AI a sustainable technology for Africa. Studies on medical equipment infrastructure in the West African sub-region attest to among other infrastructural challenges, poor equipment preservation, poor-quality management systems and obsolete medical equipment [28–30]. Besides, they expressed their uncertainties in areas such as cyber security, safety of patient data and software corruption of AI tools. This could be addressed if the healthcare industry antedates any potential surprises and meets professional requirements for AI technology to enable uninterrupted and effectual use of the technology in diagnostic imaging [1, 10]. Participants were doubtful about the cost of the AI tools and its implementation in Africa considering the economic challenges that some African countries face. Economic challenges mostly lead to broken technologies as a result of poor maintenance, and this is likely to be the same in the case of AI as the cost of implementation and maintenance may be too expensive for underdeveloped countries, pushing them further behind in improving healthcare [27]. In contrast, one participant perceived AI as an important tool in medical imaging, as it is in other fields. AI was seen as the way forward to aid accurate diagnosis and reduce errors as well as cost of expensive manpower and studies output as indicated in some publications [1, 10, 31].

Despite AI acceptance among most of the respondents, they were, however, anxious about the probable negative impact AI would have on their workforce. They argued the potential of knowledge gap that the acceptance of AI in Africa would lead to job losses and also make radiography education programmes irrelevant. This bone of contention by the participants is not surprising as there has been significant consideration of this issue that AI will lead to mechanisation of professions and extensive replacement of the human workforce [18, 24]. Despite these fears, evidence suggests that in healthcare, to date, no one has forfeited job as a result of AI implementation; rather, AI is serving an advancement role in almost all professions. This career insecurity perception has been reported in other studies [16–18, 24, 25]. However, AI will transform radiology, but not remove radiologists [24] and for that matter radiographers whose work includes direct patient interaction [25]. A few of the participants were of the view that the whole idea of AI technology should be re-examined and that there is the urgent need to raise awareness among radiographers through education, training and continuous professional development. Radiographers had previously described their willingness to interact/communicate with the patient. This indeed can be very rewarding in routine practice. Nevertheless, this seems to be somewhat contradictory with their willingness on specific training requirements.

The authors are in agreement with the findings of a publication [27], which argued that though there are several questions to answer on AI, the technology has not come to eliminate health professionals but would rather replace those who fail to use the technology, and thus, all should be prepared for its total implementation. Although previous studies have raised several issues about AI, none suggested any uncertainties about the capability of AI to advance medical imaging practice. Herein, the impression is given that AI will only be in radiology; AI will be everywhere in medicine, admittedly probably earlier in so-called medical technical disciplines, i.e. radiology, nuclear medicine, pathology, laboratory and genetics. Even the entire electronic patient file will be almost continuously explored. Therefore, there is a need for African radiographers, imaging professional bodies, AI manufacturers/vendors, policy makers and all stakeholders including governments of various African countries to work at addressing end users’ concerns about AI in medical imaging for a successful implementation of AI in Africa.

## Limitations of the study

The main limitation was the limited number of radiographers who responded to the qualitative aspect of the survey. However, being an open-ended survey questions it was not expected that all would respond. Moreover, due to poor internet connectivity across the Africa region, and communication challenges, telephone interviews were not performed which could have assisted in probing participants further for more in-depth information on perspectives. In normal times (pre-COVID), focus groups and face-to-face interview sessions would have been employed.

## Conclusions

The participants in this study were receptive of the idea to implement AI in medical imaging practice in Africa and agreed it is an indispensable tool which needs to be embraced. However, they did not hesitate to amplify some concerns including cost of the technology, impact on the workforce, ethical and legal regulatory concerns that might arise from data insecurity. The study found that radiographers in Africa would need more education on the technology and assurance of their job security. Although the qualitative responses could be predictable as with any new technology, people have various misgivings about it. In a resource poor setting, where uptake of new technologies could be a problem due to professionals being comfortable with traditional way of doing things, there is the need to assess concerns of the radiographers in particular, to address these anxieties before these implementations are in place.

The concerns of participants in the current study cannot be ignored as there are still arguments about AI algorithm performance. For this reason, this paper argues that AI would require a lot of attention from its technical performance to address the concerns of all clients. If end user concerns like issues of probable job losses, equipment maintenance culture, training and education of personnel are not addressed particularly in the African contest, AI could do more harm than good and have no positive impact on patients and the professionals who would use it. Therefore, there is a need for African radiographers, imaging professional bodies, AI manufacturers/vendors, policy makers and all stakeholders including governments of various African countries to work at addressing end users’ concerns about AI in medical imaging for a successful implementation of AI in Africa.

## Supplementary Information

**Additional file 1**. Questionnaire.

## Data Availability

On request, data would be made available.

## Abbreviations

AI: Artificial intelligence
EFRS: The European Federation of Radiographer Societies
ISRRT: The International Society of Radiographers and Radiological Technologists
Rad: Radiographer
