# Supplementary material for: Artificial intelligence in medical imaging practice in Africa: a qualitative content analysis study of radiographers’ perspectives
Source: Insights Imaging. 2021 Jun 16;12:80. doi: 10.1186/s13244-021-01028-z (PMC8206887; doi:10.1186/s13244-021-01028-z)
Supplement: Supplementary file 1 — Additional file 1. Questionnaire. [file 13244_2021_1028_MOESM1_ESM.docx]

**Appendix 1**

**Questionnaire**

1. Are you aware of artificial intelligence (AI) technology?
2. How would you describe your satisfaction with AI coming into medical imaging?
3. How would you expect AI implemented in Africa?
4. How would AI impact your career?
5. How would AI affect the way you do things as a radiographer?
6. Any other comments
